# Jasmonic acid contributes to rice resistance against *Magnaporthe oryzae*

**DOI:** 10.1186/s12870-022-03948-4

**Published:** 2022-12-20

**Authors:** Junning Ma, Jean-Benoît Morel, Michael Riemann, Peter Nick

**Affiliations:** 1 Botanical Institute, Karlsruhe Institute of Technology, Karlsruhe, Germany; 2 PHIM Plant Health Institute, Univ Montpellier, INRAE, CIRAD, Institut Agro, IRD, Montpellier, France

**Keywords:** *Magnaporthe oryzae*, Jasmonic acid, Compatible and incompatible interaction, Salicylic acid

## Abstract

**Background:**

The annual yield losses caused by the Rice Blast Fungus, *Magnaporthe oryzae*, range to the equivalent for feeding 60 million people. To ward off infection by this fungus, rice has evolved a generic basal immunity (so called compatible interaction), which acts in concert with strain-specific defence (so-called incompatible interaction). The plant-defence hormone jasmonic acid (JA) promotes the resistance to *M. oryzae*, but the underlying mechanisms remain elusive. To get more insight into this open question, we employ the JA-deficient mutants, *cpm2* and *hebiba*, and dissect the JA-dependent defence signalling in rice for both, compatible and incompatible interactions.

**Results:**

We observe that both JA-deficient mutants are more susceptible to *M. oryzae* as compared to their wild-type background, which holds true for both types of interactions as verified by cytological staining. Secondly, we observe that transcripts for JA biosynthesis (*OsAOS2* and *OsOPR7*), JA signalling (*OsJAZ8*, *OsJAZ9*, *OsJAZ11* and *OsJAZ13*), JA-dependent phytoalexin synthesis (*OsNOMT*), and JA-regulated defence-related genes, such as *OsBBTI2* and *OsPR1a*, accumulate after fungal infection in a pattern that correlates with the amplitude of resistance. Thirdly, induction of defence transcripts is weaker during compatible interaction.

**Conclusion:**

The study demonstrates the pivotal role of JA in basal immunity of rice in the resistance to *M. oryzae* in both, compatible and incompatible interactions.

**Supplementary Information:**

The online version contains supplementary material available at 10.1186/s12870-022-03948-4.

## Background

Rice is the staple food for more than half of the world population, but drastically affected by the Rice Blast Fungus, *Magnaporthe oryzae*. The yield loss is estimated to range up to 30% of the total rice production [1], and thus poses major threats to global food security. This ascomycete pursues a hemi-biotrophic lifestyle, feeding on the living tissue to obtain nutrients during an early biotrophic stage, subsequently switching to necrotrophic growth, killing the host cells by toxins and degrading enzymes. The infection cycle begins with spore attachment to the leaf surface, where spores germinate to develop a specialised penetration structure, the appressorium. The appressorium can develop, by means of osmolyte accumulation, considerable pressure to rupture cuticle and cell wall. After penetration, invasive hyphae form to acquire nutrients from the host cell, while effectors silence the immunity of the host. After 1 week of colonisation, the pathogen sporulates, such that spores can spread to neighbouring plants to initiate a new infection cycle.

In response to pathogen infection, plants have evolved a variety of strategies to protect themselves from invasion by pathogens. The first tier is formed by preformed defence, including constitutive physical or chemical barriers, such as cuticles [2], constitutively produced phytoalexins, called phytoanticipins [3, 4], or constitutive expression of defence genes [5]. Generally, preformed defence is not specific for a certain pathogen, but rather confers a broad and durable resistance to a variety of pathogens [5]. In addition to this preformed defence, plants also can rely on induced defence. The first layer of induced defence is so called pathogen-associated molecular pattern triggered immunity (PTI), activated by conserved molecular features of microbial pathogens, termed pathogen- or microbe-associated molecular patterns (PAMPs or MAMPs). For instance, detection of flagellin allows to sense most bacterial invaders, while detection of chitin allows detection of fungal attacks [6–8]. In consequence of co-evolutionary adaptation to certain pathogens, this basal layer of plant immunity can be silenced by effectors from specialised pathogens, which in turn, can activate a second layer of induced defence, called effector-triggered immunity (ETI), which is often, but not always, accompanied by hypersensitive cell death of the infected cell [9].

The complexity of innate immunity requires extensive signalling to deploy responses appropriate for the respective defence context. Plant hormones are therefore key regulators of plant immunity [10]. The biosynthesis of JA starts from the release of linolenic (18:2) and α-linolenic (18:3) acid from the chloroplast membrane, providing substrates for the 9-lipoxygenases (9-LOX) and 13-lipoxygenases (13-LOX) which catalyse the formation of 9S-hydroxyoctadecadienoic acid (HPODE) and 13S-HPODE [11]. In rice, the HPODEs are then converted by allene oxide synthase (*OsAOS1* and *OsAOS2*), and allene oxide cyclase (*OsAOC*) into 12-oxo phytodienoic acid (12-OPDA) [12, 13]. Subsequently, OPDA is shipped out of the chloroplast by a channel protein JASSY to peroxisomes [14]. Following reduction by 12-oxo phytodienoic acid reductase (*OsOPR7*) and 3 sequential steps of β-oxidation, OPDA is transformed into JA [15]. JA is further activated via the GH3 amido synthetase *OsJAR1* by conjugation of JA to the amino acid isoleucine, leading to the formation of JA-Ile [16, 17]. JA-Ile can bind to the receptor complex SCF^COI1^ to direct the degradation of JAZ proteins by way of the 26S-proteasome pathway, which can derepress downstream transcription factors, such as *OsMyc2*, and subsequent activation of JA-dependent gene expression [18, 19]. Among the 15 JAZ-encoding genes in rice, almost all of them are stress-responsive, with 9 of them particularly responsive to mechanical wounding [20].

JA and its derivatives, collectively termed as jasmonates, are reported to be involved in wounding, regulation of secondary metabolism, and the responses to abiotic and biotic stress. In dicots, jasmonates are generally responsible for defence against necrotrophic pathogens, while salicylic acid is more important for resistance against biotrophic pathogens. There exists a pronounced antagonism between jasmonate-dependent and the SA-dependent pathways, confirmed for 17 plant species so far and, thus, representing an evolutionary ancient acquisition [21]. However, the situation seems different for rice, because, here, JA and SA signalling are synergistic in most cases, although a few examples for mutual antagonism have been reported as well [22, 23]. For example, more than half of genes in rice activated by SA analog benzothiadiazole can also be induced JA, while a third of them is repressed, thus responding inversely. In rice, jasmonates has been implied to contribute to resistance against various pathogens with different lifestyles, including the hemibiotrophic pathogens *M. oryzae* and *Xanthomonas oryzae*, the necrotrophic pathogen *Rhizoctonia solani*, and the biotrophic root knot nematode *Meloidogyne graminicola* [24–26].

With respect to *M. oryzae*, previous studies have shown a role of jasmonates for different events of the the response to virulent strains. Not only can jasmonates induce the accumulation of reactive oxygen species, but they also modulate diterpenoid and flavonoid phytoalexin production after elicitation by, for instance, CuCl_2_ and chitin [27–29]. Overexpression of *OsAOS2* enhanced the accumulation of jasmonates and transcripts for pathogenesis-related (PR) gene expression during infection with the *M. oryzae* [12]. Conversely, the JA biosynthesis deletion mutants *osjar1* (a *OsJAR1* retrotransposon insertion mutant), *cpm2*, and *hebiba* (*OsAOC* deletion mutants) displayed a higher susceptibility to the this fungus, accompanied by reduced accumulation of flavonoid phytoalexins, such as sakuranetin, while the production of diterpenoid phytoalexins was not affected [13, 30]. These findings suggest a scenario, where JA can enhance rice basal defence against *M. oryzae* through promoting oxidative burst, induction of PR genes, and accumulation of the flavonoid phytoalexin sakuranetin [12, 13, 27]. However, data where jasmonate synthesis seems to be dispensable or even negatively correlated with defence against *M. oryzae* challenge this hypothesis. For example, the *OsF78i* mutant affected in the expression two omega-3 fatty acid desaturases, *OsFAD7* and *OsFAD8*, leading to marked reduction in 18:3 linolenic acid and, as a consequence, to impaired wounding-induced JA accumulation, is not more susceptible, but more resistant to *M. oryzae* [31]. Likewise, two JA knock-down mutants, *AOCRi* and *OPRRi*, deficient in JA accumulation, develop disease symptoms to the same extent as wild type plants during both, compatible and incompatible, interactions, suggesting that JA is dispensable for the defence against *M. oryzae* [32]. Even worse, a recent finding that *M. oryzae* produces jasmonates by itself to promote appressorium formation challenges the role of JA as promoting factor against *M. oryzae* [33].

We have previously identified two JA-deficient mutants, *cpm2* and *hebiba*, lacking a functional gene for Allene Oxide Cyclase (AOC) which allowed to address the role of host-derived JA for the infection process [13]. Here, we saw a difference between compatible interactions (where the mutants behaved similar to the wildtype) and incompatible interactions (where the mutants were more susceptible). These findings, using detached leaf sheaths as experimental model, indicate a possible role for JA for the hypersensitive response, as characteristic feature of incompatible interactions. There exists considerable controversy with respect to the role of JA for the hypersensitive reaction, with both, positive [34–37], and negative [38, 39] interactions being proposed. This might be partially due to differences in experimental models and conditions. The conclusions from our previous analysis is limited by the fact that excised leaf sheaths were used rather than entire living plants. Furthermore, the virulent and avirulent *M. oryzae* strains used in that study differed with respect to their genetic background. Thus, the role of jasmonates in resistance to *M. oryzae* has remained inconclusive, contrasting with the situation for bacterial blight resistance [40, 41], spikelet development [42] and drought stress [43], their function in rice blast fungus defence, where *JAZ* genes have been shown to be involved. A second open question is the interaction of JA and SA in this context [22, 44, 45].

To address these limitations of our previous study, we now used isogenic strains of *M. oryzae* carrying the cognate avirulence/resistance genes avrPia/Pia, and we conducted these studies in intact plants to more specifically explore and complete our picture on the role of JA in the interaction between rice and the pathogenic fungus *M. oryzae*. To get insight into the interaction between JA and SA signalling, we monitored not only responses jasmonate synthesis and signalling genes, but also of genes that are known to be SA responsive. We test the hypothesis that, first, JA confers defence response in both compatible and incompatible interactions, and, second, that JA-dependent defence is paralleled by a second pathway not depending on JA, but possibly linked with SA signalling during both types of interactions. To this end, we used a factorial design with two isogenic strains GY11-EV (compatible) and GY11-AvrPia (incompatible) in combination with three genotypes (Nihonmasari wildtype, *cpm2* and *hebiba*) to examine the role of JA in the defence to *M. oryzae*, through physiological, histological, and molecular approaches.

## Material and methods

### Plant material and growth condition

In this study, we compared three rice genotypes (Nihonmasari WT, *cpm2* and *hebiba*) that were a collection of plant materials in our lab and generated as described previously [13]. The mutants *cpm2* and *hebiba* are JA-deficient mutants arose by γ-irradiation in the background of the *O. sativa ssp. japonica* cultivar Nihonmasari and are affected in the synthesis of jasmonates due to deletions in the gene for *allene oxide cyclase (AOC)*. The *cpm2* mutant harbours a 11-bp deletion in exon 1 of AOC, while in the *hebiba* mutant, a large deletion of 170 kb eliminated not only the entire AOC, but also neighbouring genes [13]. Since homozygous mutants are male sterile, we had to maintain the lines through the heterozygotes, from which we were able to select homozygous plants due to their growth phenotype (the coleoptiles show an altered light response and therefore are long under irradiation, while the heterozygotes or the wild type seedlings show short coleoptiles). Seeds of good quality from each genotype were selected and surface-sterilised with 80% ethanol for 1 min, and then rinsed two times with sterile water, followed by soaking in 5% sodium hypochlorite for 20 min. We removed hypochlorite residues by rinsing the seeds with sterile water three times. The sterilised seeds were sown onto 0.45% phytoagar gel filled in Magenta boxes and allowed to germinate under a 12 h: 12 h light: dark cycle (photosynthesis active radiation (PAR) 120 μmol m^− 2^ s^− 1^). We selected seedlings displaying the phenotype (long coleoptiles) as homozygous mutants. The selected mutants and seedlings displaying the wild-type phenotype were subsequently transplanted to pots filled with compost (7/8 Neuhaus compost no. 9, and 1/8 Pozzolana, Neuhaus, France). The soil was humidified every day and fertilised once weekly (1.5 g/L NPK (17–7-22), and 0.25 g/L QUELARTAL Fe (6% w/v, Artal, Valencia). After further 14 days of growth in the greenhouse (20 °C during the night, and 30 °C during daytime), the seedlings had reached the four-leaf stage and were ready for inoculation.

### Cultivation of *Magnaporthe* strains and infection assay

We employed two genetically engineered strains of *Magnaporthe oryzae* for the current study [46]: strain GY11-EV, harbouring an empty vector was virulent for the rice cultivar Nihonmasari, while the engineered strain-GY11-AvrPia expressed the avirulence gene *AvrPia*, and consequently was avirulent on Nihonmasari. Both strains were cultivated on rice flour medium and fungal spores harvested [47]. We adjusted the concentration of the suspension to 5 × 10^4^ spores/ml with 0.5% gelatine for inoculation. Following inoculation, the plants were incubated in a dew chamber (25 °C, 100% humidity) in darkness for 16–18 h, and then transferred to a growth chamber (12 h in darkness at 25 °C and 12 h in fluorescent light at 30 °C, photosynthetically available radiation 120 μmol m^− 2^ s^− 1^) for the development of the symptoms. At days 1, 2 and 3 post inoculation (dpi), we sampled the fourth leaf of each plant for histological analysis or RNA extraction. At 7 dpi, when symptoms had fully appeared, infected leaves were excised, pasted onto a sheet of scale paper, and scanned digitally. The images were then subsequently used for quantitative image analysis using ImageJ (imagej.nih.gov/ij), scoring lesion areas (Analyze/Tool/ROI Manager), lesion number (Plugins/Analyze/Cell counter), and total leaf area (Analyze/Tool/ROI Manager). The total coverage of lesion areas, as well as the number of lesions were then normalised to total leaf area.

### Histological analysis of fungal growth in rice leaves

Infected leaves were sampled at 2 and 3 dpi by cutting the fourth leaf into approximately 4 cm long segments, followed by fixation in 75% (v/v) ethanol, 25% (v/v) chloroform, and 0.15% (v/v) acetic acid for 2 days until the chlorophyll was fully removed. Subsequently, we cleared the tissue by rinsing with distilled water twice for 15 min, and then macerating with 0.05 M NaOH at 90 °C in a water bath for 15 min. Subsequently, we washed away NaOH with distilled water twice for 10 min, followed by buffering the tissue with Tris-HCl (0.1 M, pH 5.8) at 90 °C in a water bath for 30 min. After washing out the buffer with distilled water for 10 min, the tissue was pre-equilibrated with sterilized Phosphate Buffered Saline (PBS, pH 7.4) for 15 min at 20 °C, before staining with 0.002% WGA-Alexa Fluor 488 (Invitrogen, USA) in PBS overnight at 20 °C in darkness. We kept the stained tissue in sterilised 50% (v/v) glycerol at 4 °C until microscopic observation. Fungal hyphae in rice leaf tissue were observed under an epifluorescence microscope (AxioImager Z.1 microscope, Zeiss, Jena) equipped with an ApoTome microscope slider for optical sectioning and a cooled digital CCD camera (AxioCamMRm) using filter set 38 HE (excitation at 470 nm, beam splitter at 495 nm, and emission at 525 nm). To follow fungal development in the tissue, we constructed frequency distributions over the developmental stages sampling at least 100 individual specimens.

**Fig. 1:**
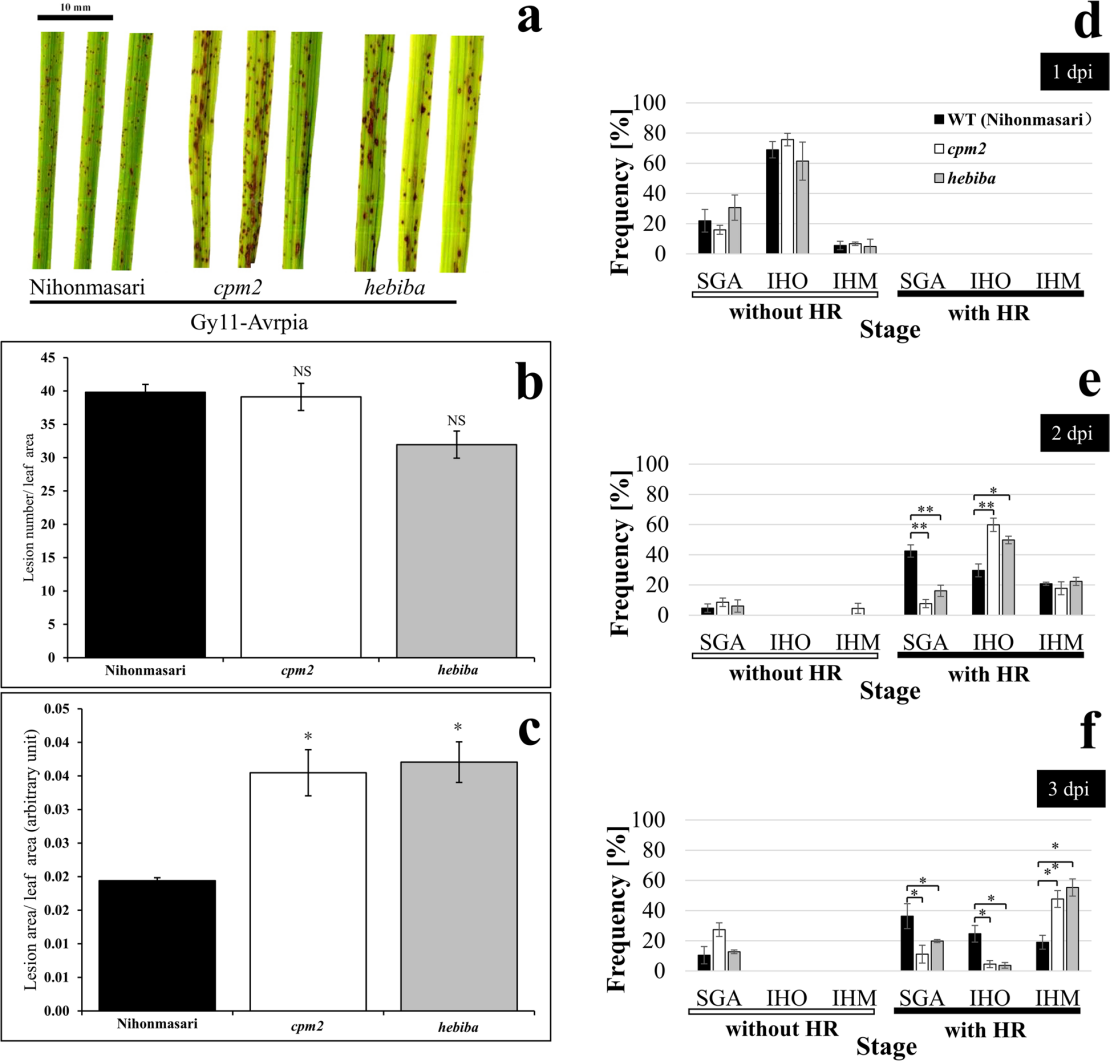
Response of rice to the avirulent strain Gy11-AvrPia. **a**-**c** Representative symptoms and quantification on forth leaf blade from three genotypes of rice: Nihonmasari WT, *cpm2*, and *hebiba* at 7 dpi. **a**: representative leaves showing the level of symptoms; **b**: lesion number per leaf area (lesion number/cm^2^); **c**: lesion area per leaf area. For each genotype, leaves from at least 3 seedlings in different pots were collected for symptom quantification. **d**-**f** Quantification of fungal development during incompatible interaction with strain GY11-AvrPia rice leaf using the staging system of Fig. S1. Frequency distributions of the different stages at 1 dpi (**d**), 2 dpi (**e**), and 3 dpi (**f**). Error bars indicate standard error of three biological replicates. “NS” indicates that no significant difference was found as compared to Nihonmasari WT (*P* > 0.05, Student’s *t*-test). Significant differences are indicated by * (*P* < 0.05, Student’s t-test), or ** (*P* < 0.01, Student’s t-test), respectively

**Fig. 2:**
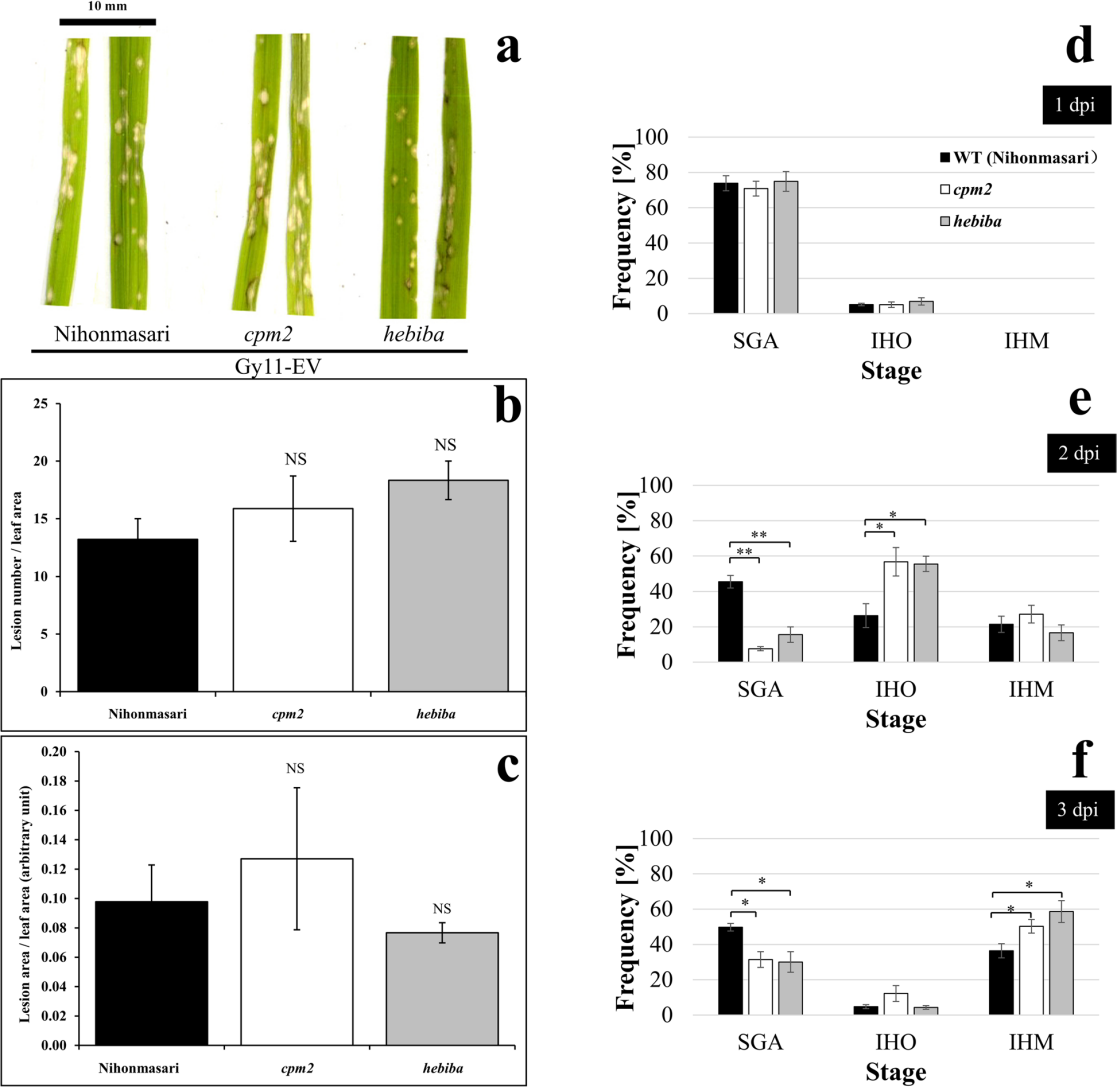
Response of rice to the avirulent strain Gy11-EV. **a**-**c** Representative symptoms and quantification on forth leaf blade from three genotypes of rice: Nihonmasari WT, *cpm2*, and *hebiba* at 7 dpi. **a**: representative leaves showing the level of symptoms; **b**: lesion number per leaf area (lesion number/cm^2^); **c**: lesion area per leaf area. For each genotype, leaves from at least 3 seedlings in different pots were collected for symptom quantification. **d**-**f** Quantification of fungal development during compatible interaction with strain GY11-EV rice leaf using the staging system of Fig. S1. Frequency distributions of the different stages at 1 dpi (**d**), 2 dpi (**e**), and 3 dpi (**f**). Error bars indicate standard error of three biological replicates. “NS” indicates that no significant diffenrence was found as compared to Nihonmasari WT (*P* > 0.05, Student’s *t*-test). Significant differences are indicated by * (*P* < 0.05, Student’s t-test), or ** (*P* < 0.01, Student’s t-test), respectively

As to have a closer look at fungal growth within the plant tissue prior to full expression of symptoms with both GY11-EV virulent strain and GY11-AvrPia avirulent strain, samples from 1, 2 and 3 dpi were used for histological analysis. To get a quantitative approach, fungal growth was classified into the following categories: S non-germinated spore, SG germinated spore, SGA germinated spore with appressorium, IHO invasive hyphae in one cell, and IHM invasive hyphae in multiple cells (Fig. S1). In addition to the classification of the pathogen, the response of the host tissue in the neighbourhood of the spore was classified with respect to absence or presence of a hypersensitive reaction (Fig. S1). It has to be mentioned that stage S (ungerminated spores) and SG (germinated, but not attached) were not considered in quantification of fungal development, because these stages are mostly lost during the staining procedure (Figs. 1d-f and 2d-f).

### Inducibility of defence-related genes by jasmonate

To investigate the inducibility of defence-related genes by jasmonate, we sprayed 100 μM (approximately 5 ml for each seedling) of Methyl Jasmonic Acid (MeJA) including 0.5% gelatine, or as mock solution, 0.5% gelatine only, to wildtype Nihonmasari seedlings at the four-leaf stage, raised as mentioned above. The fourth leaf was excised after 24 h of incubation and immediately frozen in liquid nitrogen for RNA extraction and gene expression analysis.

### RNA extraction, cDNA synthesis and real-time qPCR

RNA was extracted from leaf samples of both, MeJA application assay at 24 hpi as well as the *Magnaporthe* infection assay at 2 and 3 dpi were used in this procedure, reversely transcribed into cDNA, and used to measure steady-state transcript levels of target genes (Supplementary Table S1) by Real-Time qPCR (CFX-96, BioRad, Munich) as described previously [48]. The genes for transcript analysis were selected on the principle that these genes were either associated with jasmonate biosynthesis and signalling, or related to defence and reported to be regulated by jasmonate. In the case of jasmonate signalling genes, selection of only five JAZ genes for the analysis was established on a pre-screening test in which these genes were more pronouncedly induced by mechanical wounding (data not shown). The expression of the genes was normalised to the expression of glycerinaldehyde-3-phosphate-dehydrogenase (*GAPDH*) as housekeeping gene as per the 2^−ΔΔCT^ method [49]. The stability of house-keeping gene in expression across all the samples was validated in a preliminary test by comparison with three alternative house-keeping genes, coding for two ubiquitins (*UBI5, UBI10*), and one actin (*ACT11*) as described in [50]. To minimise the technical variation between samples and measurements in the qPCR assay, we adopted a strategy, where all samples were run on the same plate and also the reference gene was always measured in the same run with the genes of interest. For each treatment, three biological replicates were conducted, each in 2–3 technical replicates, to obtain robust results. For accession numbers, description of gene functions as well as primer sequences of the genes measured in this study see Supplementary Table S1.

### Statistical analysis

We conducted the statistical analysis using the add-in Real Statistics Resource Pack (http://www.real-statistics.com/free-download/real-statistics-resource-pack/) for Excel. We used a homoscedastic, two-tailed t-test to test differences in disease severity and fungal growth between the JA-deficient mutants and the Nihonmasari wild type, where significant differences (*P* < 0.05) are indicated by *, and highly significant difference (*P* < 0.01) with **. For the multiple comparison of different transcript levels between any pair of treatments during compatible and incompatible interactions, we employed a One-Way ANOVA followed by Duncan’s test using the R package Agricolae, where significant differences (*P* < 0.05) are indicated by different letters above each bar. In the case of histology and transcript analysis, we conducted three biological replicates (in technical triplicates). For each biological replicate, we pooled segments from 3 to 4 individual seedlings. For the histological analysis, we investigated segments from 2 to 3 individual seedlings per experiment.

## Results

### During incompatible interaction, jasmonate mutants showed stronger symptoms and faster colonisation

To validate the previously reported [13] phenotype of the JA-mutants with respect to incompatible interaction, we inoculated with the avirulent strain GY11-AvrPia of *M. oryzae*. Here, the wild type Nihonmasari was clearly more resistant compared to *cpm2* and *hebiba*: in both mutants, lesions covered almost twice the area as compared to the wild type (Fig. 1c). In contrast, there was no significant difference as for the lesion number per leaf area among the three genotypes (Fig. 1b), indicative of a situation, where not the initiation of a lesion, but its expansion differed between mutants and wild type.

Since the individual spores are only loosely connected with the host tissue prior to appressoria formation, they are mostly washed off during the staining procedure. It did not make sense, therefore, to include these early stages into the quantitative analysis. Thus, we could only score the stages SGA, IHO, and IHM in a reliable manner (Fig. S1). When we followed colonisation by the avirulent strain GY11-AvrPia over time, we saw a clear progression through colonisation stages. Specifically, the initial phase (1 dpi) did not reveal any difference between Nihonmasari and the two mutants (Fig. 1d): the majority of attached spores were in the state of appressorium formation (stage SGA), only few had proceeded to invade one cell (stage IHO). The frequency of these two stages were also identical to those seen for the virulent strain GY11-EV (Fig. 2d), indicating that strain differences of the host response were not manifest at this early stage. This changed drastically during the subsequent days. From 2 dpi, we observed a distinct hypersensitive reaction for the avirulent strain (Fig. 1e), manifest from appressorium formation. Infected cells without a hypersensitive reaction were almost absent – no matter, whether in the wild type or the two mutants, which remained true also for 3 dpi (Fig. 1f). However, the progression from the appressorium stage into the subsequent stages (hyphae invading individual or multiple cells) was swifter and more pronounced in amplitude in the two mutants, as compared to the wild type (Fig. 1e and f). Thus, colonisation was more successful in the mutants, resembling the situation seen for the virulent strain GY11-EV (Fig. 2e and f). However, the avirulent strain exhibited a significantly higher difference between wild type and mutants, albeit a hypersensitive reaction was also seen in the mutants.

### During compatible interaction, jasmonate biosynthesis mutants showed similar symptoms, but faster colonisation

The situation was slightly different in case of compatible interaction, when we inoculated with the strain GY11-EV. Here, symptom expression in Nihonmasari wild type, and the two jasmonate deficient mutants *cpm2* and *hebiba* did not display significant differences **(**Fig. 2a, b and c) as reflected also by the quantitative indicators (lesion number / leaf area and lesion area / leaf area). Specifically, the number of lesions in the *cpm2* mutant was mildly (by around 25%), that in the *hebiba* mutant more substantially (by almost 50%) increased over the number in the wild type, but in none of the mutants was the difference significant (Fig. 2b). Also, for the coverage of lesion area, there was no significant difference (Fig. 2c). Taken together*,* susceptibility in *cpm2* and *hebiba* to the virulent strain GY11-EV was comparable to their wild-type background Nihonmasari. For the cytological analysis in compatible interaction, only the stages SGA, IHO, and IHM (Fig. S1) were scored for the purpose of reliability due to the same reason as described above. When we followed colonisation by the virulent strain GY11-EV over time, we saw a clear progression through subsequent stages: while at 1 dpi most individuals just had formed appressoria (stage SGA, Fig. 2d), at 2 dpi many had invaded individual cells (stage IHO, Fig. 2e), and at 3 dpi many had proceeded to infect multiple cells (stage MHO, Fig. 2f). It should be noted that stages SGA and IHO represent steady states between increase by spores that germinated with some delay and decrease by progression to the next stage. While colonisation proceeded in both wild type and mutant hosts, there were specific differences: In the wild type, from those individuals that had formed appressoria at 1 dpi, around half had invaded the host cells at 2 dpi (roughly half of those just one cell, the other half multiple cells). The other half remained arrested at the stage of appressoria formation. This conclusion is based on the fact that, at 3 dpi, still half of the population had not advanced beyond the appressoria stage, while the sum of stages IHO and MHO had remained the same as 2 dpi. In other words, almost 60% of individuals in stage SGA had not contributed to the subsequent stages during the time interval between 2 dpi and 3 dpi. For the two jasmonate-synthesis mutants, the progression from appressoria formation to the invasion of individual cells (2 dpi) was significantly accelerated, which also holds true for the invasion into neighbouring cells (3 dpi). In contrast to the wild type, there was also a significant recovery in the frequency of cells in stage SGA at 3 dpi as compared to 2 dpi (Fig. 2e, f), indicative of a second wave of appressoria coming from hitherto non-germinated spores. Thus, invasion of virulent GY-EV strain into cells and progression into neighbouring cells proceeded more swiftly in hosts that are not able to activate jasmonate signalling.

### Inducibility of defence-related genes by MeJA

To test the inducibility of defence-related genes (based on their reported inducibility by *Magnaporthe* infection) by jasmonate, we treated wild-type rice with 100 μM of MeJA and scored the induction of steady-state transcript levels at 24 h after inoculation (Fig. S2). The tested genes were selected from four categories, namely jasmonate biosynthesis genes (*OsAOS1*, *OsAOS2*, *OsAOC*, *OsOPR7* and *OsJAR1*), jasmonate signalling genes (*OsJAZ8*, *OsJAZ9*, *OsJAZ10*, *OsJAZ11* and *OsJAZ13*), defence-related genes (*OsPR1a*, *OsPR1b*, *OsBBTI2*, *OsPBZ1*, *OsCPS2* and *OsCPS4*), and genes involved in phenylpropanoid pathway (*OsPAL1*, *OsNOMT*, *OsCAD2* and *OsCOMT1*). The specific function of the genes is detailed in the subsequent sections. Most transcripts showed mild inductions below one order of magnitude. However, some transcripts showed a more prominent induction: *OsJAZ8* was induced 25-fold, *OsJAZ11*, *OsBBTI2*, and *OsNOMT* around 15-fold. The genes were highly responsive to MeJA, thus being good candidate genes for following transcript analysis in jasmonate-deficient mutants. Even though some other genes are reported to be highly induced by *Magnaporthe* infection, their inducibility by MeJA was not as significant as expected. For instance, defence-related genes such as *OsPR1a*, *OsPR1b*, *OsPBZ1*, *OsCPS2* and *OsCPS4* are key players positively regulated by jasmonate signalling, but their transcripts were only slightly or hardly affected by MeJA treatment (Fig. S2). A similar induction pattern was also found for other jasmonate biosynthesis, jasmonate signalling genes, and for phenylpropanoid pathway genes. Nevertheless, this does not preclude that these genes might show a transient response already declined at the time point of scoring transcripts. Since the responsiveness to MeJA might not completely correlate with the responsiveness to *Magnaporthe* infection, all selected genes would be still used for subsequent transcription analysis for infected wild type and jasmonate mutants.

### Jasmonate synthesis: *OsAOS2* is a key gene during incompatible interaction

Since it was clear that the JA deficient mutants were less resistant to the *M. oryzae* as compared to their wildtype background ‘Nihonasari’, we were probing for potential differences in genes expression correlated to differential resistance. As both mutants were affected in the locus for the JA-biosynthesis gene allene oxide cyclase (AOC), we measured the response of JA biosynthesis genes.

**Fig. 3:**
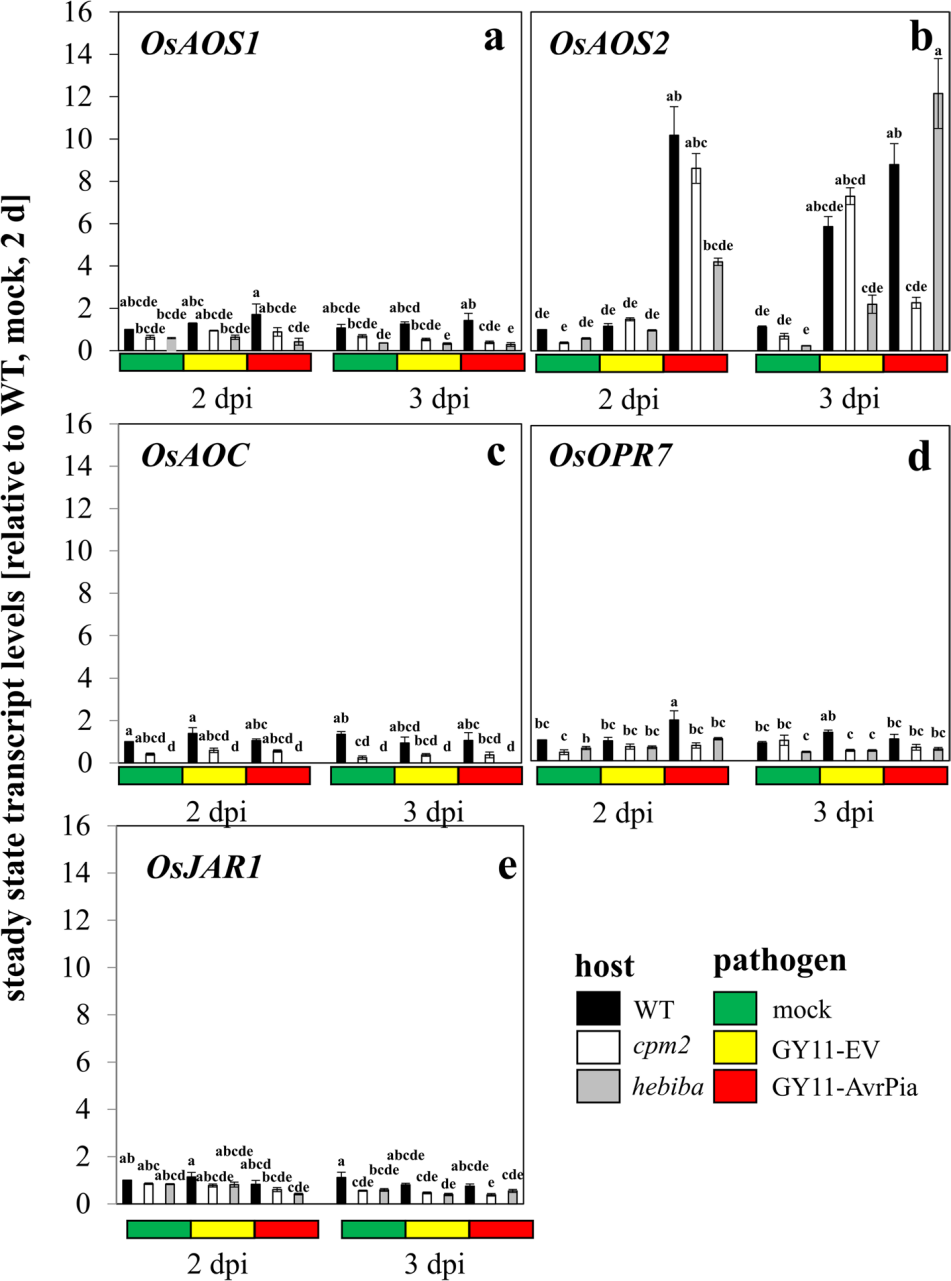
Steady-state transcript levels for genes involved in jasmonate biosynthesis in response to mock treatment, or inoculation with the compatible strain GY11-EV, or the incompatible strain GY11-AvrPia in WT and the two jasmonate biosynthesis mutants. **a**: *OsAOS1*; **b**: *OsAOS2*; **c**: *OsAOC*; **d**: *OsOPR7*; **e:** *OsJAR1*. Data represent mean values and standard errors relative to the value measured at 2 d, mock control in the WT. Comparison of transcript level between any pair of treatments was conducted using One-Way ANOVA followed by a Duncan’s test. Significant difference (*P* < 0.05) was indicated by different letters on each bar. Data represent three independent experimental series with three technical replications per experiment

The steady-state transcript levels of *OsAOS1*, *OsAOS2*, *OsOPR7*, *OsAOC* and *OsJAR1* were generally lower in *cpm2* and *hebiba*, as compared to the wild type (Fig. 3). Moreover, the response of these transcripts to fungal inoculation was almost non-existent. There was one exception, though: *OsAOS2* was induced rapidly (from 2 dpi) and strongly (around 10 folds) during incompatible interaction with GY11-AvrPia in the wild type (Fig. 3b). This distinctive expression pattern of *OsAOS2* was also validated by hierarchical clustering in a heatmap, where all the jasmonate biosynthesis genes except *OsAOS2* were clustered in one group (Fig. S3a). In *cpm2*, this response was slightly reduced in amplitude (around 8 folds at 2 dpi) and less stable (at 3 dpi it had already dropped to around 2-fold (as compared to more than 8-fold in the wild type). For *hebiba*, the pattern was different. Here, the induction was delayed, but with an amplitude similar to the wild type. For compatible interaction with GY11-EV, we saw the response as well, but with a delay by 1 day and did not reach the same amplitude. For the wild type, an induction of around 6-fold was seen for wild type, but also for *cpm2*, while it was only 2-fold in *hebiba*. Thus, induction of *OsAOS2* occurs specifically during fungal infection. This induction is stronger during incompatible interaction. In the two mutants, it occurs with reduced amplitude (*cpm2*) or initiates later (*hebiba*).

### Jasmonate signalling: *OsJAZ9* is the key gene during incompatible interaction

**Fig. 4:**
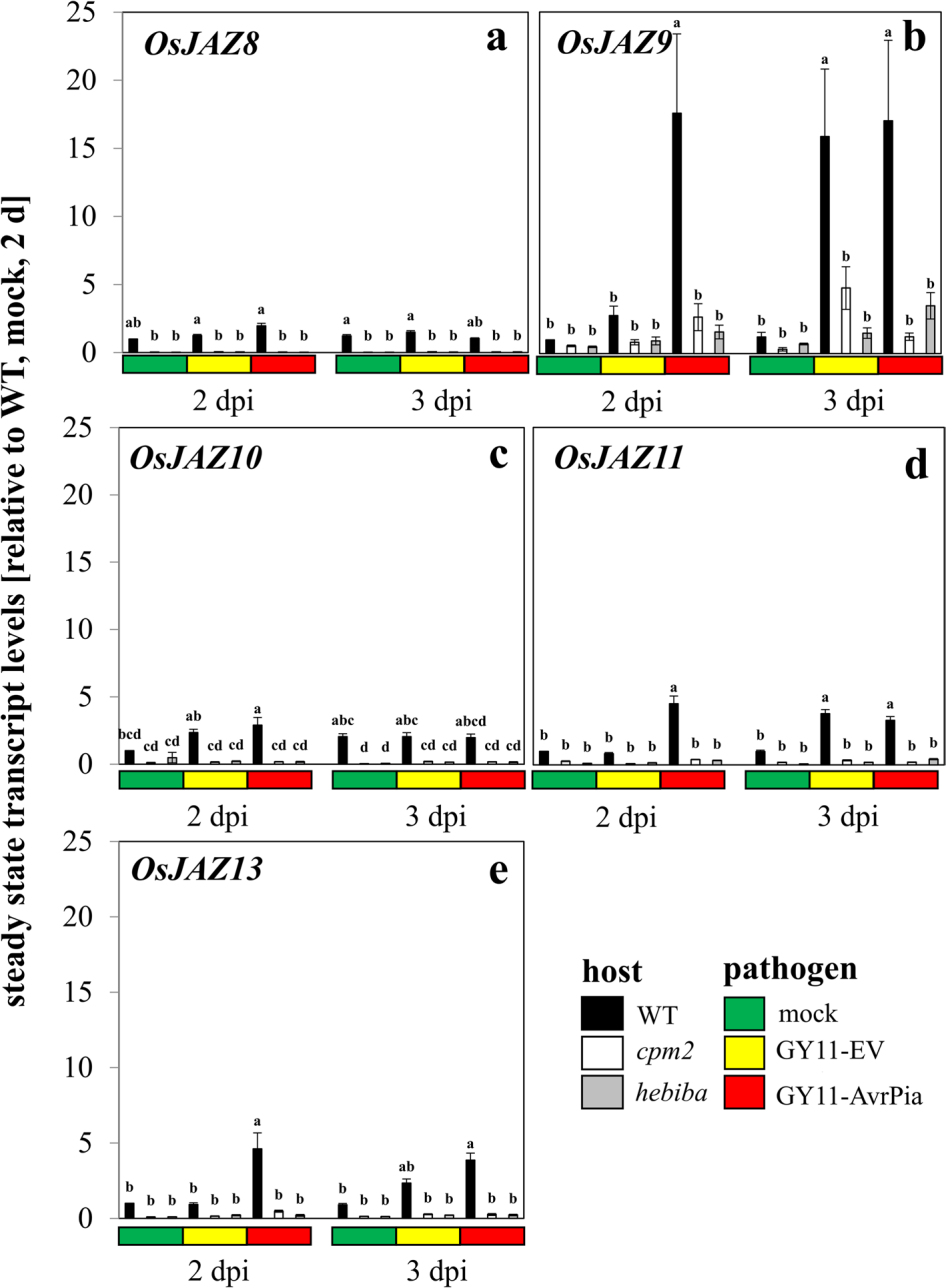
Steady-state transcript levels for genes involved in jasmonate signalling in response to mock treatment, or inoculation with the compatible strain GY11-EV, or the incompatible strain GY11-AvrPia in WT and the two jasmonate biosynthesis mutants. **a** *OsJAZ8*; **b** *OsJAZ9*; **c** *OsJAZ10*; **d** *OsJAZ11*; **e** *OsJAZ13*. Data represent mean values and standard errors relative to the value measured at 2 d, mock control in the WT. Comparison of transcript level between any pair of treatments was conducted using One-Way ANOVA followed by a Duncan’s test. Significant difference (*P* < 0.05) was indicated by different letters on each bar. Data represent three independent experimental series with three technical replications per experiment. Nomenclature follows Ye et al. (2009)

Since the jasmonate deficient mutants were impaired in their defence against the fungus, we wondered if the transcription of genes involved in jasmonate response like *OsJAZ* could be impaired. Hereby, we scrutinised *OsJAZ8*, *9*, *10*, *11* and *13*, because they had been reported as wound-inducible [30]. In the beginning, we also considered *OsJAZ12*, but did not pursue it later. The reason was a non-steady melting curve of the qPCR amplification, indicating inhomogeneities. Therefore, the readout was not reliable. Unfortunately, the nomenclature of rice *JAZ* genes is not standardised. For the sake of clarity, we have therefore decided to follow the nomenclature given in [20]. Generally speaking, the expression pattern of *JAZ* genes was clearly clustered in two groups in hierarchical clustering analysis in which *JAZ9* was separated from other *JAZ* genes (Fig. S3b). Specifically, except for *JAZ9*, the transcript responses of other *JAZ* genes were of minor amplitudes (in the range of 1 to 5-fold), but still it became evident that both mutants were accumulating significantly lower steady-state levels of *OsJAZ8* (Fig. 4a), *OsJAZ10* (Fig. 4c), *OsJAZ11* (Fig. 4d), and *OsJAZ13* (Fig. 4e) as compared to the wild type. These relatively sluggish responses contrasted sharply with the pattern observed for *OsJAZ9* (Fig. 4b). Here, the wild type reached much higher levels (more than 15-fold as compared to the control). Like the pattern observed for *OsAOS2*, the accumulation of *OsJAZ9* transcripts became manifest earlier (already at 2 dpi) for the incompatible interaction with GY11-AvrPia, while for the compatible interaction with GY11-EV, this accumulation was reached only 1 day later. The mutants did respond as well, however, the amplitudes where not comparable (remaining below 5-fold induction, Fig. 4b), which is in stark contrast to the situation seen for *OsAOS2*, where the mutants did reach up to comparable transcript levels as the wild type (Fig. 3b). However, ignoring these differences in relative amplitude, the temporal patterns of *OsAOS2* and *OsJAZ9* transcription were comparable as validated in Fig. S3c. In *cpm2*, the response of *OsJAZ9* was seen already at 2 dpi (albeit with low amplitude) and found to decline, if scored 1 day later, while for hebiba, the response was still low at 2 dpi, but did increase rather than decline during the following day. The same temporal pattern had been seen in case of *OsAOS2*, however, the responses of JAZ9 in the mutants where much lower, although significant. This leads to the conclusion that *OsJAZ9* is induced specifically during fungal infection. This induction is faster during incompatible interaction, and it was strongly impaired in the jasmonate biosynthesis mutants.

### Jasmonates negatively regulate the induction of *OsPR1b* and *OsCPS2*

In the next step, we probed the expression of a panel of defence-related genes reported to be jasmonate responsive, to get insight into specific downstream pathways: The pathogenesis-related protein *OsPR1a* belongs to the acidic PR proteins, reported to be upregulated by MeJA [51]. In parallel, *OsPR1b* encoding the most prominent member of basic PR proteins, was probed, for which the literature record with respect to JA responsiveness was discrepant (no induction by 100 μM MeJA reported by [51], strong induction by 100 μM JA by [12]. In a third report, the inducibility of *OsPR1a* and *OsPR1b* by JA or SA or *Magnaporthe* had been d found only in the old, but not the young leaves of rice [52].Also, the transcript encoding the Bowman-Birk protease inhibitor *BBTI2* [23], and the *OsPR10a / PBZ1* coding for a RNase, both reported as JA inducible [53], were included into the study. In addition, we addressed two transcripts for *ent*-copalyl diphosphate synthases, *OsCPS2* and *OsCPS4*, because they are involved in the formation of labdane-related diterpenoids, discussed as phytoalexins [54]. In the same work, a knockdown of *OsCPS2*, but not of *OsCPS4* caused a modulated response to *M. oryzae*. Both genes respond to the jasmonate pathway [55].

**Fig. 5:**
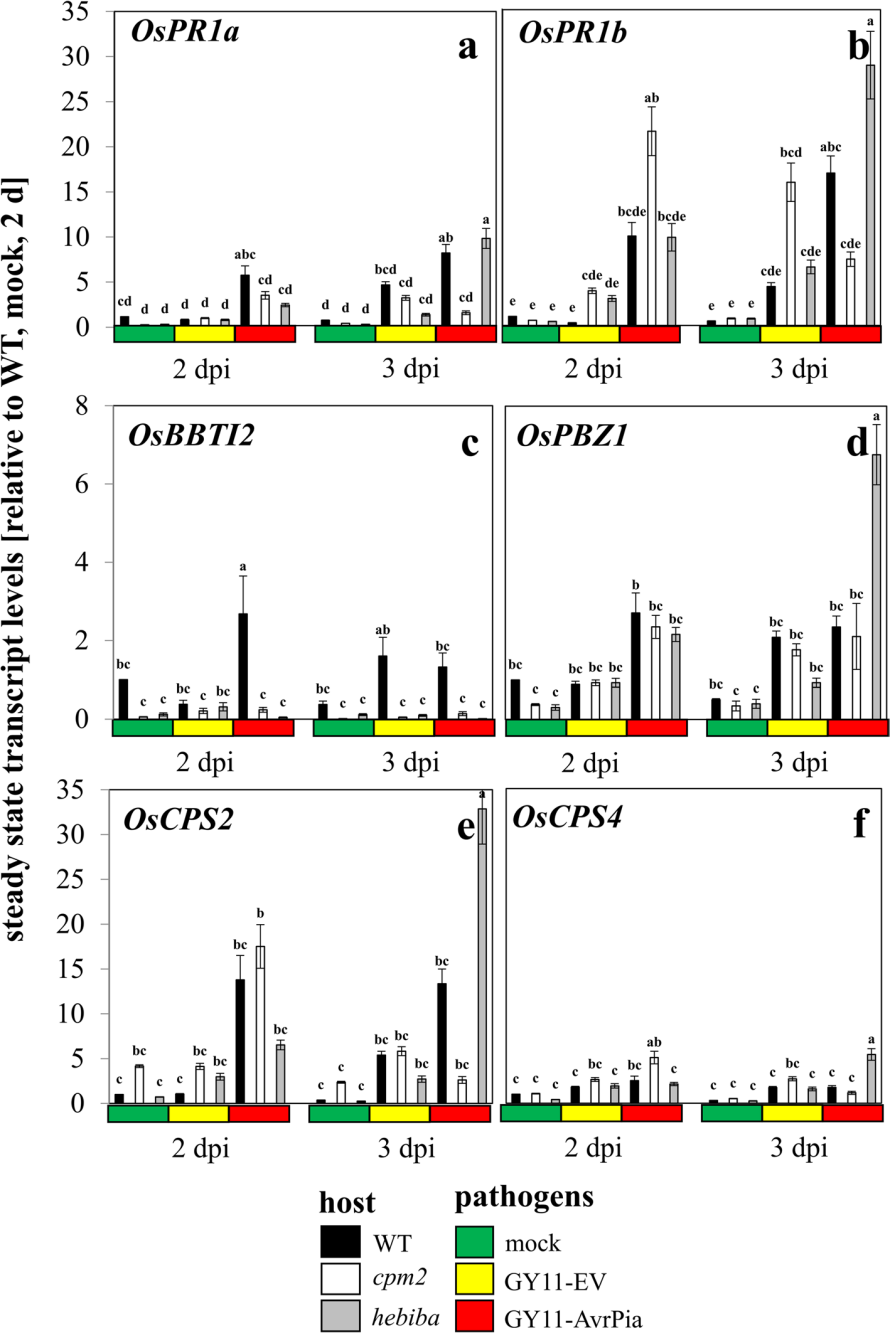
Steady-state transcript levels for jasmonate responsive defence genes in response to mock treatment, or inoculation with the compatible strain GY11-EV, or the incompatible strain GY11-AvrPia in WT and the two jasmonate biosynthesis mutants. **a** *OsPR1a*; **b** *OsPR1b*; **c** *OsBBTI2*; **d** *OsPBZ1*; **e** *OsCPS2*; **f** *OsCPS4*. Data represent mean values and standard errors relative to the value measured at 2 d, mock control in the WT. Comparison of transcript level between any pair of treatments was conducted using One-Way ANOVA followed by a Duncan’s test. Significant difference (*P* < 0.05) was indicated by different letters on each bar. Data represent three independent experimental series with three technical replications per experiment

The resulting patterns (Fig. 5) were clearly specific for the type of gene, the type of interaction (compatible or incompatible), and for the host genotype. Overall, *OsPR1b* and *OsCPS2* sticked out specifically among the tested jasmonate-responsive transcripts responsive to *M. oryzae*. This was based on two observations: (1) both transcripts showed a more substantial accumulation in mutants, indicative of a negative regulation by jasmonates; (2) both transcripts showed an earlier, but transient amplification in *cpm2*, while the induction in *hebiba* initiates later, but is more persistent thereafter, indicating that in addition to the impaired allene oxide cyclase activity other genetic factors absent in *hebiba* (but still present in *cpm2*) played a role in the regulation of *OsPR1b* and *OsCPS2* genes (Fig. 5b and e). The distinct role of *OsPR1b* and *OsCPS2* was also verified in the hierarchical clustering analysis, with the cluster of *OsCPS2* and *OsPR1b* being clearly separated from other genes (Fig. S3d). Specifically, *OsPR1b* encoding a basic PR protein, known as strongly inducible during *Magnaporthe* infection, was rapidly and transiently induced during incompatible interaction, with a delay as well as a lower amplitude during compatible interaction (Fig. 5b), while *OsCPS2* encoding an *ent*-copalyl diphosphate synthase displayed a similar and strong induction pattern (Fig. 5e). Thus, the transcript of *OsPR1b* and *OsCPS2* shared the following pattern details – earlier induction during incompatible interaction, amplified response in the mutants as compared to the wild type, swifter, and transient response in *cpm2*, delayed but stronger response in *hebiba* (Fig. 5b and e). In contrast, transcription patterns of *OsPR1a* (encoding an acidic PR protein) and *OsBBTI2* (a Bowman-Birk protease inhibitor) clustered more closely, both not being differently induced in the compatible interaction between WT and mutants until later stage of infection (namely 3 dpi), but earlier and significantly diverging during the incompatible interaction between WT and mutants (Fig. 5a and c). The induction of *OsPR1a* and *OsBBTI2* seemed to require jasmonate biosynthesis, since it was absent in both mutants. The response of *PBZ1* (Fig. 5d) was of a comparatively minor amplitude, again initiating earlier during incompatible interaction. Here, the mutants were not significantly different from the wild type, with one surprising exception: during day 3 of the incompatible interaction, there was a strong upregulation in case of *hebiba* as a host, while *cpm2* only produced the same induction seen in the wild type. Similarly, the induction of *OsCPS4* (coding for another *ent*-copalyl diphosphate synthase) was of a relatively low amplitude in both compatible and incompatible interactions, and was found significantly higher in mutants (either *cpm2* or *hebiba*) than wild type (Fig. 5f).

### Jasmonates are required for the activation of a phytoalexin synthesis gene

**Fig. 6:**
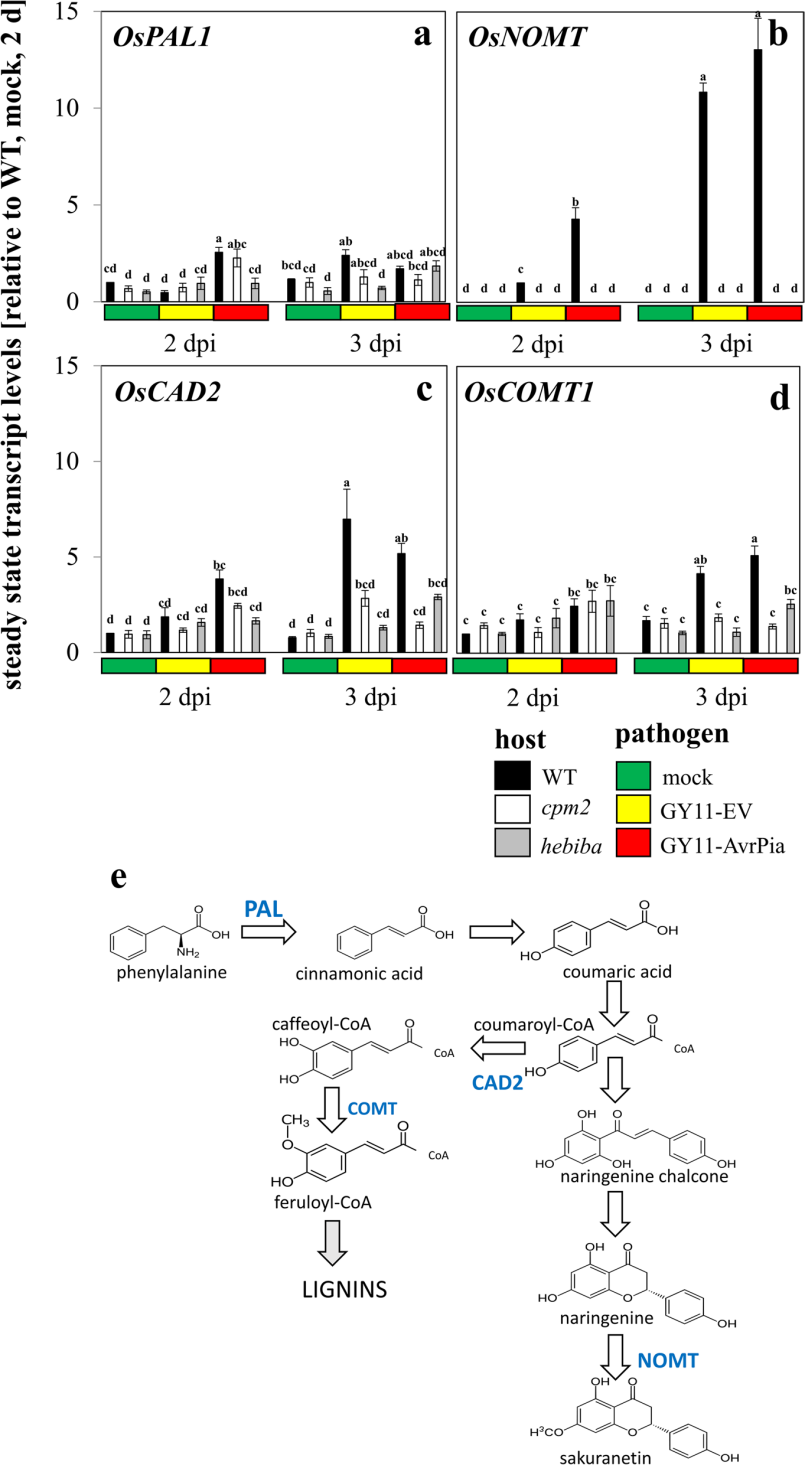
Steady-state transcript levels for genes of the phenylpropanoid pathway in response to mock treatment, or inoculation with the compatible strain GY11-EV, or the incompatible strain GY11-AvrPia in WT and the two jasmonate biosynthesis mutants. **a** *OsPAL1*; **b** *OsNOMT*; **c** *OsCAD2*; **d** *OsCOMT1*; **e** the pathway indicating the site of action. Data represent mean values and standard errors relative to the value measured at 2 d, mock control in the WT. Comparison of transcript level between any pair of treatments was conducted using One-Way ANOVA followed by a Duncan’s test. Significant difference (*P* < 0.05) was indicated by different letters on each bar. Data represent three independent experimental series with three technical replications per experiment

The accumulation of antifungal defence compounds (phytoalexins) represents an efficient response against invasion. Many plants accumulate phenolic compounds by activation of the phenylpropanoid pathway. In rice, the flavonoid sakuranetin [56] is one of the most relevant phytoalexins. We therefore probed several genes of the phenylpropanoid pathway (Fig. 6e), such as phenyl ammonium lyase (PAL) as first committed step of secondary metabolism, along with naringenin 7-O-methyltransferase (NOMT), the enzyme that gives rise to sakuranetin. To probe for the activity of the monolignol branch of the phenylpropanoid pathway, the transcripts for cinnamyl alcohol dehydrogenase 2 (CAD2), the first committed step of monolignol biosynthesis in rice, along with those for caffeic acid O-methyltransferase (COMT) catalysing the subsequent step giving rise to ferulic acid were measured (Fig. 6e). In the wild type, transcripts for PAL (Fig. 6a) showed only a minute, but significant induction by around twofold. This induction occurred more swiftly in case of incompatible interaction. This induction was also present in *cpm2*, but delayed in *hebiba*. Instead, there was a clear and strong response for the *NOMT* transcripts (Fig. 6b), exceeding one order of magnitude at day 3 and developing more swiftly during incompatible as compared to compatible interaction. This response was completely absent from the mutants indicating that the response of *NOMT* to infection is strictly dependent on jasmonates. The two tested transcripts representing the monolignol branch were induced (Fig. 6c and d), albeit to a lesser extent (around 5-fold), and with a less strict dependence on jasmonates, because, here, the two mutants still were able to produce a partial response. Thus, *NOMT* as phytoalexins-synthesis gene clearly sticks out among the four tested candidates of the phenylpropanoid pathway as the transcript with the strongest response. The point was also confirmed in the hierarchical clustering, with *NOMT* as an independent cluster among the four phenylpropanoid metabolism related genes (Fig. S3e). It was also the transcript with the strictest dependence on jasmonates.

## Discussion

Our motivation for the current work was to compare the role of the jasmonate pathway for the resistance against and the response to virulent or avirulent strains of *M. oryzae*. The strains were isogenic and differed only with respect to the cognate avirulence/resistance genes avrPia/Pia. This allowed to address, more specifically and independently of the pathogen background, incompatible and compatible interactions.

We observe that the jasmonate-deficient mutants, *cpm2* and *hebiba*, develop stronger symptoms and allow a faster progression of fungal development. This enhanced susceptibility in mutants correlates with the specific lack of induction of transcripts for *OsAOS2* (JA biosynthesis), *OsJAZ9* (JA signalling), and *OsNOMT* (JA-dependent phytoalexin synthesis), especially for incompatible interaction. The fact that JA can suppress some of the defence-related genes, such as *OsPR1b*, *OsCPS2*, and that infection and exogenous methyl jasmonate induce different patterns of transcript accumulation indicate that fungal interaction triggers additional pathways in parallel to jasmonate synthesis and signalling. In the following, we will discuss to what extent the jasmonate pathway can orchestrate defence against *M. oryzae*, whether compatible and incompatible interaction differ in this respect, and what other signals might be involved.

### Jasmonate signalling is necessary for defence against *M. oryzae*

Both jasmonate-depletion mutants (*cpm2* and *hebiba*) showed a more pronounced expression of symptoms (Fig. 1a, b and c) and a faster colonisation of host cells (Fig. 1d, e and f) upon infection in the incompatible interaction. This was consistent with previous observations conducted on detached leaf sheath [13] and extends the phenomenon to entire living plants. Moreover, the faster or more extended invasion (Fig. 1d, e and f) can also explain the larger visible necrosis (Fig. 1a) observed in both mutants upon initiation of hypersensitive response. This is also congruent with the finding that *osjar1* mutant (Tos17 insertion mutant affected in the conjugation of isoleucine and, thus, deficient in JA-Ile) was more susceptible than its parental wild type, as well as displaying significantly bigger necrotic lesions during incompatible interaction [30]. Even though the visual difference was not reaching significance in the case of compatible interaction (Fig. 2a, b and c), when we scrutinised the stages of infection (Fig. 2d, e and f), we saw that the transition from formation of an appressorium towards invasion of the first cell represented a bottleneck in case of the wild type, while this step proceeded more swiftly in the mutants. This is in line with the report that the jasmonate over-accumulation restrains fungal growth during compatible interaction compared to the wild type situation [12]. This cytological difference between wild type and jasmonate mutants was independent of the type of interaction, because we saw a similarpattern for both, compatible (Fig. 2e and f**)** and incompatible interaction (Fig. 1e and f), where, especially at 2 dpi, a hypersensitive response of the host cell ensued (Fig. 1). Only at 3 dpi, differences between interaction types emerged, when more than 20% of infection events were arrested in the first cell (IHO stage) for incompatible interaction, in contrast to less than 5% in compatible interaction. However, still fungal colonisation remained more efficient in both mutants compared to the wild type (Figs. 2f and 3f). Altogether, the jasmonate-deficient mutants (*cpm2* and *hebiba*) were impaired in defence against *M. oryzae* in both, incompatible and compatible, interactions.

For the wild type, the delay in the transition from appressorium formation to invasion into the first host cell came with a specific induction of transcripts for *OsJAZ9* (Fig. 4b), *OsAOS2* (Fig. 3b) and *OsNOMT*, the key enzyme for the phytoalexin sakuranetin (Fig. 6b). The response of *OsNOMT* was completely absent in the mutants, irrespectively of the type of interaction, indicative of an absolute requirement for jasmonate synthesis. The induction of *OsJAZ9* was mostly, but not entirely absent in the mutants, reporting a strong but not exclusive dependence on jasmonates. In contrast, the induction of *OsAOS2* was not eliminated, but just delayed, suggesting that a jasmonate-independent pathway can convey the signal, but requires jasmonates to proceed swiftly. The fact that *OsJAZ9* and *OsAOS2* are partially under control of a jasmonate-independent signal is also consistent with the map of MeJA responsiveness (Fig. S2), where both transcripts were not prominent with respect to induction. This is contrasting with *OsNOMT*, which exhibited a conspicuous response to MeJA.

**Fig. 7:**
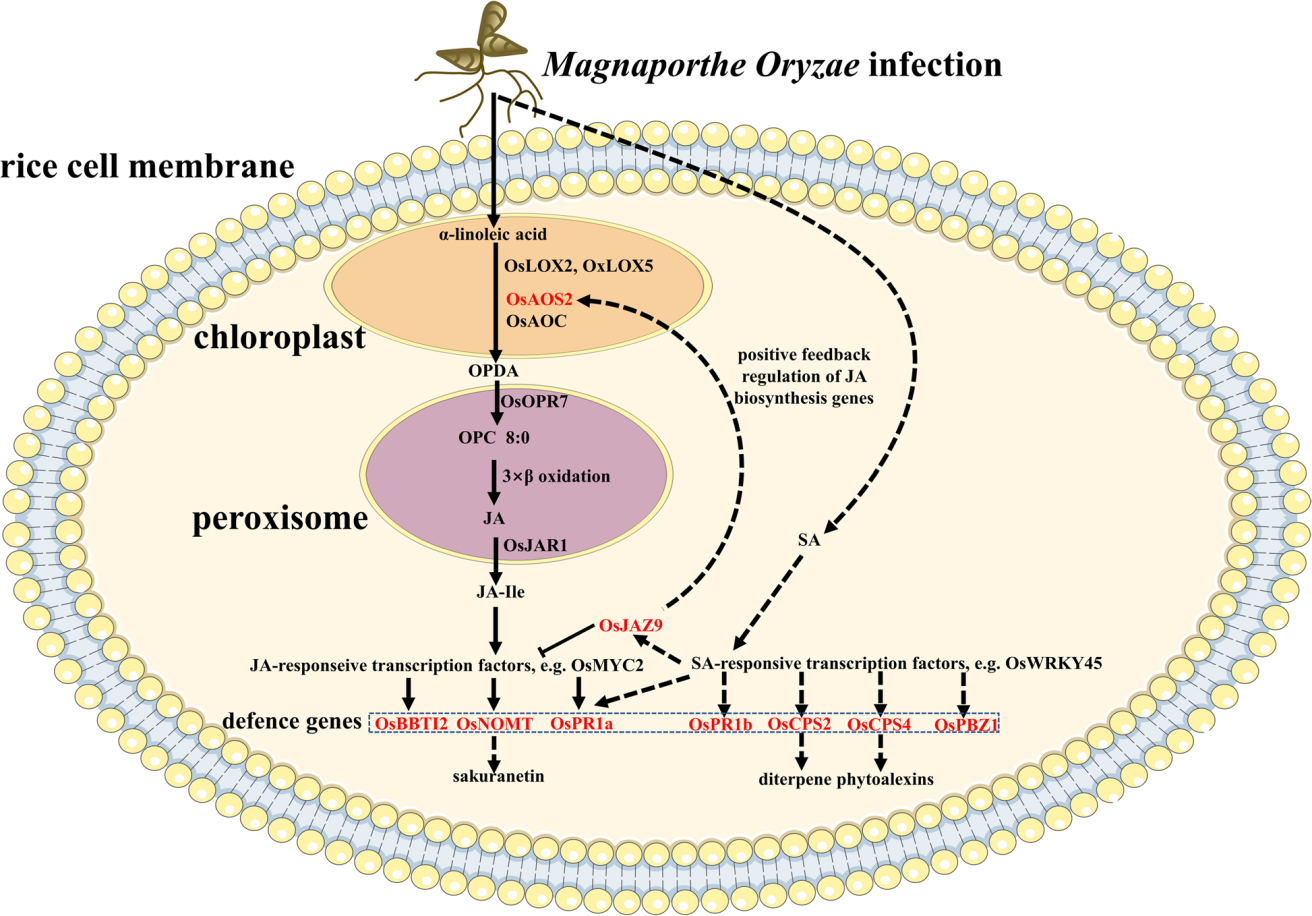
Schematic diagram illustrating the model of JA-dependent and JA-independent pathway in response to *M. oryzae* infection. Note: solid lines in graph indicate JA-dependent pathway examined in this study, whereas dashed lines represent the speculative JA-independent pathway (possibly through SA-dependent signalling pathway) that was not verified in this study

These findings lead to a model (Fig. 7), where infection with *M. oryzae* activates jasmonate synthesis and sakuranetin accumulation (probably through *OsJAZ9*), which helps to impair the step from appressorium formation to hyphal penetration. In parallel, a jasmonate-independent event activates *OsAOS2* in concert with positive feedback from jasmonate signalling. This mechanism is active for both fungal strains but proceeds more swiftly when the interaction is incompatible.

The published record has already demonstrated important implications of this model. For instance, the UV-response of *OsNOMT* is strictly dependent on bioactive jasmonates. The *osjar1* mutant is not able to conjugate isoleucine to jasmonate, and likewise fails to induce *OsNOMT* in response to UV [57]. Likewise, the key role of *OsAOS2* is in line with a study, where overexpression of *OsAOS2* enhanced the induction of the defence-genes *PR1a*, *PR3* and *PR5* upon infection in compatible interaction [12]. A candidate for the feedback of jasmonate signalling on *OsAOS2* expression might be specific WRKY factors. In *Arabidopsis*, herbivory through *Spodoptera exigua* results in a calcium dependent phosphorylation of the JAV1-JAZ8-WRKY51 protein complex that can de-repress *AOS* genes [58]. Specificity on the level of JAZ-proteins is also in line with the finding that resistance of rice to the bacterial pathogen *Xanthomonas oryzae pv. oryzae* is regulated by a different member (*OsJAZ8*) of this family [41].

### *OsAOS2* and *OsJAZ9* are two key players in JA-dependent pathway

While the majority of jasmonate-synthesis related transcripts were not very responsive to infection, *OsAOS2* was clearly behaving differently as also validated by hierarchical clustering (Fig. S3a). During incompatible interaction, this transcript increased strongly and swiftly (Fig. 3c), while this response occurred 1 day later during compatible interaction. The two mutants were able to deploy this response, albeit at somewhat reduced amplitude (*cpm2*) or at a later time point (*hebiba*), suggesting a pivotal role of *OsAOS2* during incompatible interaction. The fact that overexpression of *OsAOS2* in rice (causing elevated jasmonate levels) led to elevated resistance against a moderately virulent strain of *M. oryzae* [12] would support the notion that *OsAOS2* is also one of the factors underlying basal immunity in compatible interaction. A straightforward hypothesis would be that the ability to potently induce jasmonate biosynthesis is hall marker for strong basal immunity in both compatible and incompatible interactions. If this holds true, one would expect that jasmonate synthesis or stability are target for pathogen effectors. This is consistent with the fact that the virulent strain of *M. oryzae* GY11 uses the microRNA *miR319* to quell the JA biosynthesis genes *OsLOX2* and *OsLOX5* [59]. Likewise, virulent strains of *M. oryzae* can secrete a monooxygenase that converts JA in rice plant to 12OH-JA (inactive in JA defence signalling), thereby weakening basal immunity and facilitating colonisation in host [60]. Moreover, exogenous JA increased the resistance to a virulent strain of *M. oryzae* [13]. In addition, the induction of *OsAOS2* seems to proceed via a JA-independent pathway (as we can conclude that it was also present in the mutants), albeit JA seems to promote this pathway (as we can conclude from the lower or later response in the mutants).

In contrast, *OsJAZ9*, acting as crucial jasmonate response factor during defence to *M. oryzae* (Fig. S3b), was strictly depending on JA, since this response was completely absent in the mutants (Fig. 4b). Its transcription pattern in response to *M. oryzae* infection was clustered together with *OsAOS2* among the jasmonate biosynthesis and signalling genes, pointing to that *OsJAZ9* is also regulated in the same pathway as that activating *OsAOS2*. Similarly, its response was 1 day earlier during incompatible as compared to compatible interaction, consistent with the hypothesis that fungal effectors (such as *miR319*) [59] silence JA biosynthesis genes or activate JA catabolism [60] to delay host basal immunity. Taken together, *OsJAZ9* is another critical player in combination with *OsAOS2* in potentiation of basal immunity.

### SA might be involved in the *OsJAZ9*-independent pathway

Among potential downstream genes, some show different patterns, where the induction in jasmonate-deficient mutants is stronger than in the wild type, indicative of involvement of an *OsJAZ9*-independent pathway. In this context, the strong and specific response of the acidic PR protein *OsPR1b* (Fig. 5b) is worth mentioning because it increased more strongly JA mutants, in both compatible and incompatible interactions. Although this gene is responsive to exogenous jasmonates [12, 61], it remains responsive in the mutants as well, and is even inducible to a higher degree. Thus, this response does not require jasmonates and seems to run independently of *OsJAZ9*. Since *OsPR1b* can also be strongly induced by salicylic acid, suggesting that this second pathway involving salicylic acid can sometimes act in synergy with jasmonate signalling in terms of *M. oryzae* resistance [21, 51]. A similar conclusion was reached for *OsCPS2*. This was also confirmed in hierarchical clustering analysis in which *OsPR1b* and *OsCPS2* clustered differently from *OsJAZ9* (Fig. S3f), suggesting that the *OsJAZ9*-independent pathway was involved in regulating *OsPR1b* and *OsCPS2*. Similar conclusions derive from the patterns for *OsPBZ1* (Fig. 5d) and *OsCPS4* (Fig. 5f) that, in certain cases, are more responsive in the mutants as well. Both genes have been reported to be independent of JA [62, 63], again corroborating a role for *OsJAZ9*-independent signalling. A candidate for such a SA-dependent pathway would be signalling through *OsWRKY45*. In fact, the SA analog benzothiadiazole was shown to prime rice plants to rapidly upregulate *OsCPS2* and *OsCPS4* transcripts and subsequent diterpene phytoalexin production in response to *Magnaporthe* infection in a *OsWRKY45*-dependent manner [63].

### *OsNOMT* is a marker gene of the JA-dependent pathway

In addition to the transcript with the most pronounced JA-dependence, *OsNOMT* (Fig. 6b), also the transcript of the Bowman-Birk trypsin inhibitor *OsBBTI2* responded more swiftly during incompatible interaction in a strict dependence on jasmonates, as evident from the lacking induction in the mutants (Fig. 5c). Further support comes from the strong response of this transcript to exogenous MeJA (Fig. S2), which was more conspicuous than the induction upon infection. This inhibitor, in concert with heat shock transcription factor HSF23, mediates constitutive defence against *M. oryzae* in rice [64]. Even though the transcription of *OsPR1a* displayed a certain JA-dependence as well, this was less strict since the transcript level during incompatible interaction at 3 dpi were comparable between *hebiba* and wildtype (Fig. 5a). Since *hebiba* harbours multiple gene deletions extending beyond the lack of *OsAOC*, the difference to *cpm2* indicates that JA is not the main player here. This is also congruent with the publishedrecords, where *OsPR1a* has been reported to be strongly inducible in response to SA and H_2_O_2_, and moderately by abscisic acid [65]. The same holds true for *OsCAD2* (Fig. 6c) and *OsCOMT1* (Fig. 6d). Both transcripts were positively correlated with JA levels in most cases (less induced but not abolished in JA mutants), indicating a role in JA-dependent signalling in this specific scenario. Both genes encode enzymes for lignin biosynthesis in rice, which might be relevant as physical barrier to pathogen penetration [66–68]. Altogether, it is *OsNOMT* that qualifies as best marker gene for JA signalling, followed to some extent by *OsBBTI2*.

### Outlook - is SA the second player during incompatible interaction?

Both mutants display necrotic lesions that extend beyond the degree seen in the wildtype (Fig. 1a). This allows two conclusions. First, PCD seems to proceed independently of jasmonate signalling. Second, JA might be a negative regulator of PCD itself, or, more likely, impair cell-cell spread of the pathogen.

The literature record on the role of JA in PCD is quite discrepant, to put it mildly. In some cases, it seems to attenuate PCD by antagonistic crosstalk with SA-mediated PCD [35, 38] or constraining concurrent 9-LOX derived oxylipin signalling [69–71]. In other cases, JA enhances stress induced PCD by synergy with SA signalling [36, 37]. This discrepancy may stem from the fact that the functional context matters and that possible different types of PCD are involved [72].

To get more insight in the role of salicylic acid, we are currently analysing its interaction with jasmonate dependent signalling, because this interaction might be a target of co-evolution between *M. oryzae* and its host, *O. sativa.*

Both JA mutants displayed significant difference from the wild type with respect to the cytological aspects of both, compatible and incompatible interactions, especially at 2 dpi. Likewise, as discussed above, the activation of *OsAOS2* at 2 dpi most likely involves factors that are independent of JA. Even though WRKY transcription factors qualify as potential candidates, we still need to address this experimentally, for instance, by conducting a transcriptomic analysis during the early stages of response (1 dpi or even earlier) in order to identify specific factors responsible for the observed increase of susceptibility in jasmonate-deficient mutants and for the induction of *OsAOS2*.

## Supplementary Information

**Additional file 1: Table S1.** Primers used in this experiment for real-time PCR. **Fig. S1.** Representative images to illustrate the staging system used to classify the colonisation of plant tissue by including both strains GY11-EV and GY11-Avrpia. a-e. without host HR response; f-h. with host HR response. a. Spore without germination. b. Spore with germination; c. Spore with germ tube and appressorium formation but without host HR response; d. Invasive hyphae in one cell without host HR response; e. Invasive hyphae in multiple cells without host HR response; f. Spore with germ tube and appressorium formation with host HR response; g. Invasive hyphae in one cell with host HR response; h. Invasive hyphae in multiple cells with host HR response. Sp spore; Ap appressorium; IH invasive hypha; SGA germinated spore with appressorium; IHO invasive hyphae in one cell; IHM invasive hyphae in multiple cells. **Fig. S2.** Steady-state transcript levels for genes of jasmonate biosynthesis, jasmonate signalling, defence, and phenylpropanoid metabolism in response to 200 μM of MeJA, scored 24 hours after the onset of the treatment in leaves of the wildtype. The heat map shows the fold-induction over the mock treatment. **Fig. S3.** Hierarchical clustering of the transcript levels for genes of jasmonate biosynthesis (**a**), signalling (**b**), both jasmonate biosynthesis and signalling (**c**), defence (**d**), phenylpropanoid metabolism (**e**), *OsAOS2* + *OsJAZ9* + defence+phenylpropanoid metabolism (**f**) and all tested genes (**g**) in response to mock treatment, or inoculation with the compatible strain GY11-EV, or the incompatible strain GY11-AvrPia in WT and the two jasmonate biosynthesis mutants. Note: numbers for the heatmap row names represent the following: **1**: 2 dpi-mock-WT, **2**: 2 dpi-mock-*cpm2*, **3**: 2 dpi-mock-*hebiba*, **4**: 2 dpi-GY11-WT, **5:** 2 dpi-GY11-*cpm2*, **6**: 2 dpi-GY11-*hebiba*, **7**: 2 dpi-GY11-AvrPia-WT, **8:** 2 dpi-GY11-AvrPia-*cpm2*, **9**: 2 dpi-GY11-AvrPia-*hebiba*, **10**: 3 dpi-mock-WT, **11**: 3 dpi-mock-*cpm2*, **12**: 3 dpi-mock-*hebiba*, **13**: 3 dpi-GY11-WT, **14:** 3 dpi-GY11-*cpm2*, **15**: 3 dpi-GY11-*hebiba*, **16**: 3 dpi-GY11-AvrPia-WT, **17:** 3 dpi-GY11-AvrPia-*cpm2*, **18**: 3 dpi-GY11-AvrPia-*hebiba*.

## Data Availability

The data used and analyzed in this study are available from the corresponding author on reasonable request.
