# Supplementary material for: Jasmonic acid contributes to rice resistance against Magnaporthe oryzae
Source: BMC Plant Biol. 2022 Dec 20;22:601. doi: 10.1186/s12870-022-03948-4 (PMC9764487; doi:10.1186/s12870-022-03948-4)
Supplement: Supplementary file 1 — Additional file 1: Table S1. Primers used in this experiment for real-time PCR. Fig. S1. Representative images to illustrate the staging system used to classify the colonisation of plant tissue by including both strains GY11-EV and GY11-Avrpia. a-e. without host HR response; f-h. with host HR response. a. Spore without germination. b. Spore with germination; c. Spore with germ tube and appressorium formation but without host HR response; d. Invasive hyphae in one cell without host HR response; e. Invasive hyphae in multiple cells without host HR response; f. Spore with germ tube and appressorium formation with host HR response; g. Invasive hyphae in one cell with host HR response; h. Invasive hyphae in multiple cells with host HR response. Sp spore; Ap appressorium; IH invasive hypha; SGA germinated spore with appressorium; IHO invasive hyphae in one cell; IHM invasive hyphae in multiple cells. Fig. S2. Steady-state transcript levels for genes of jasmonate biosynthesis, jasmonate signalling, defence, and phenylpropanoid metabolism in response to 200 μM of MeJA, scored 24 hours after the onset of the treatment in leaves of the wildtype. The heat map shows the fold-induction over the mock treatment. Fig. S3. Hierarchical clustering of the transcript levels for genes of jasmonate biosynthesis (a), signalling (b), both jasmonate biosynthesis and signalling (c), defence (d), phenylpropanoid metabolism (e), OsAOS2 + OsJAZ9 + defence+phenylpropanoid metabolism (f) and all tested genes (g) in response to mock treatment, or inoculation with the compatible strain GY11-EV, or the incompatible strain GY11-AvrPia in WT and the two jasmonate biosynthesis mutants. Note: numbers for the heatmap row names represent the following: 1: 2 dpi-mock-WT, 2: 2 dpi-mock-cpm2, 3: 2 dpi-mock-hebiba, 4: 2 dpi-GY11-WT, 5: 2 dpi-GY11-cpm2, 6: 2 dpi-GY11-hebiba, 7: 2 dpi-GY11-AvrPia-WT, 8: 2 dpi-GY11-AvrPia-cpm2, 9: 2 dpi-GY11-AvrPia-hebiba, 10: 3 dpi-mock-WT, 11: 3 dpi-mock-cpm2, 12: 3 dpi-mock-h [file 12870_2022_3948_MOESM1_ESM.docx]

**Jasmonic acid contributes to rice resistance against *Magnaporthe oryzae***

**Junning Ma^1^, Jean-Benoît Morel^2^, Michael Riemann^1^, Peter Nick^1*^**

^1^ *Botanical Institute, Karlsruhe Institute of Technology, Karlsruhe, Germany.*

^2^ *PHIM Plant Health Institute, Univ Montpellier, INRAE, CIRAD, Institut Agro, IRD, Montpellier, France*

***Correspondence:** **peter.nick@kit.edu; +49 721 608-42144**

**Supplementary material**

**Table S1 Primers used in this experiment for real-time PCR.**

| **Gene symbol** | **Locus ID** | **Gene description** | **Forward primer (5’-3’)** | **Reverse Primer (5’-3’)** | **Purpose** | **Reference** |
| --- | --- | --- | --- | --- | --- | --- |
| *OsJAZ8* | Os09g0439200 | Jasmonate ZIM-domain protein 8; repressor of jasmonic acid signalling | GAAGGCTCAACAGCTGACCAT | TTGGTGGACGGGAAGTTCTC | Target gene for qPCR | [1] |
| *OsJAZ9* | Os03g0180800 | Jasmonate ZIM-domain protein 9; repressor of jasmonic acid signalling | GGCCGGTCGAGTTGGAA | GGTCAGGCTCGGCGAAAT | Target gene for qPCR | [1] |
| *OsJAZ10* | Os03g0181100 | Jasmonate ZIM-domain protein 10; repressor of jasmonic acid signalling | TCTTCCCACCCCGTCAAAT | CCTCGCTGGTGCTTTGCT | Target gene for qPCR | [1] |
| *OsJAZ11* | Os03g0180900 | Jasmonate ZIM-domain protein 11; repressor of jasmonic acid signalling | CAGCCTTGCCTACCAGACATG | GACGATCCTGTTCTTCCTCTTCTC | Target gene for qPCR |  |
| *OsJAZ13* | Os10g0391400 | Jasmonate ZIM-domain protein 13; repressor of jasmonic acid signalling | ACACGTCAGCTTTAATCCCATAATT | GAATAATCGTGCACTGTACAAATGC | Target gene for qPCR | [1] |
| *OsAOS1* | Os03g0766900 | Allene oxide synthase 1; involved in jasmonic acid biosynthesis | CACCGTCACCTCGCTCAAGAAG | ACTCCGTATCCGTACAAGCTGATTG | Target gene for qPCR |  |
| *OsAOS2* | Os03g0225900 | Allene oxide synthase 1; biosynthesis of jasmonic acid | GGAGGAAGCTGCTGCAATAC | GGAGGTTGAAGCTTTGGTGA | Target gene for qPCR |  |
| *OsAOC* | Os03g0438100 | Allene oxide cyclase; biosynthesis of jasmonic acid | TGCCTCAACAACTTCACCAACTA | CACATGCCGCAATTAACACTAAA | Target gene for qPCR |  |
| *OsJAR1* | Os05g0586200 | Jasmonyl-L-isoleucine synthase | AGGAGGCATCAAAGTTCCTGG | CTCAGCTCCCAGAAGATCACG | Target gene for qPCR |  |
| *OsOPR7* | Os08g0459600 | 12-oxophytodienoate reductase | CTCAACCACCGGTTTCCTCA | TCCATGCATCAGTCTGCTCT | Target gene for qPCR |  |
| *GAPDH* | Os04g0486600 | Cytosolic glyceraldehyde-3-phosphate dehydrogenase 2 | AAGCCAGCATCCTATGATCAGATT | CGTAACCCAGAATACCCTTGAGTTT | Selected reference gene for qPCR | [2] |
| *UBQ10* | Os02g0161900 | Ubiquitin 10 | TGGTCAGTAATCAGCCAGTTTGG | GCACCACAAATACTTGACGAACAG | Candidate reference gene for qPCR | [2] |
| *UBQ5* | Os01g0328400 | Ubiquitin 5 | ACCACTTCGACCGCCACTACT | ACCACTTCGACCGCCACTACT | Candidate reference gene for qPCR | [2] |
| *ACT11* | AK100267 | Actin 11 | CAGCCACACTGTCCCCATCTA | AGCAAGGTCGAGACGAAGGA | Candidate reference gene for qPCR | [2] |
| *OsPR1a* | Os07g0129200 | Pathogenesis-related protein 1a | GTATGCTATGCTACGTGTTTATGC | GCAAATACGGCTGACAGTACAG | Target gene for qPCR | [3] |
| *OsPR1b* | Os01g0382000 | Pathogenesis-related protein 1b | ACGCCTTCACGGTCCATAC | AAACAGAAAGAAACAGAGGGAGTAC | Target gene for qPCR | [3] |
| *OsPBZ1* | Os12g0555000 | Root specific pathogenesis-related protein 10 | ATGAAGCTTAACCCTGCCGC | GTCTCCGTCGAGTGTGACTTG | Target gene for qPCR |  |
| *OsCPS2* | Os02g0570900 | Ent-copalyl diphosphate synthase gene 2 | CGAGGAGCTTACTGTACGC | TGAGCAGATCTCGATTGTG | Target gene for qPCR | [4] |
| *OsCPS4* | Os04g0178300 | Ent-copalyl diphosphate synthase gene 4 | GTGTTGTAGCGTTGAAGTCA | CAATCTCAAATCCAACTAGCA | Target gene for qPCR | [4] |
| *OsBBTI2* | Os01g0124000 | Similar to Bowman Birk trypsin inhibitor | CGGAGCCGCCTCGCTATATC | CTAGAACACACGCAGACTGATCG | Target gene for qPCR | [5] |
| *OsNOMT* | Os12g0240900 | Naringenin 7-O-methyltransferase, Biosynthesis of flavonoid phytoalexin sakuranetin | CTAGCCGGATGCATGAAAGT | TGCACGTATAGGCACACACA | Target gene for qPCR | [6] |
| *OsPAL1* | Os02g0627100 | Phenylalanine ammonia-lyase | GGGCAACCCAGTGACCAA | CGATTGCCTCGTCGGTCTT | Target gene for qPCR |  |
| *OsCOMT1* | Os08g0157500 | Caffeic acid O-methyltransferase | CCTCGCCCTCATGAACCA | CGTCCAGGACTGCGTCCTTA | Target gene for qPCR | [7] |
| *OsCAD2* | Os02g0187800 | Cinnamyl-alcohol dehydrogenase, involved in lignin biosynthesis | CGACCAGAAGTTTGTGGTGAA | GAAGTGCTTCAGTGGGCTGTA | Target gene for qPCR | [8] |

Note: the rationale of selecting *OsJAZ8*, *9*, *10*, *11* and *13* for analysis in this study was based on consideration that these genes had been reported as wound-inducible [9], which was also confirmed during preparatory studies, where their response to wounding was assessed. In the beginning, we also considered OsJAZ12, but did not pursue it later. The reason was a non-steady melting curve of the qPCR amplification, indicating inhomogeneities. Therefore, the readout was not reliable. Unfortunately, the nomenclature of rice JAZ genes is not standardised. For the sake of clarity, we have therefore decided to follow the nomenclature given in the puablication [10].


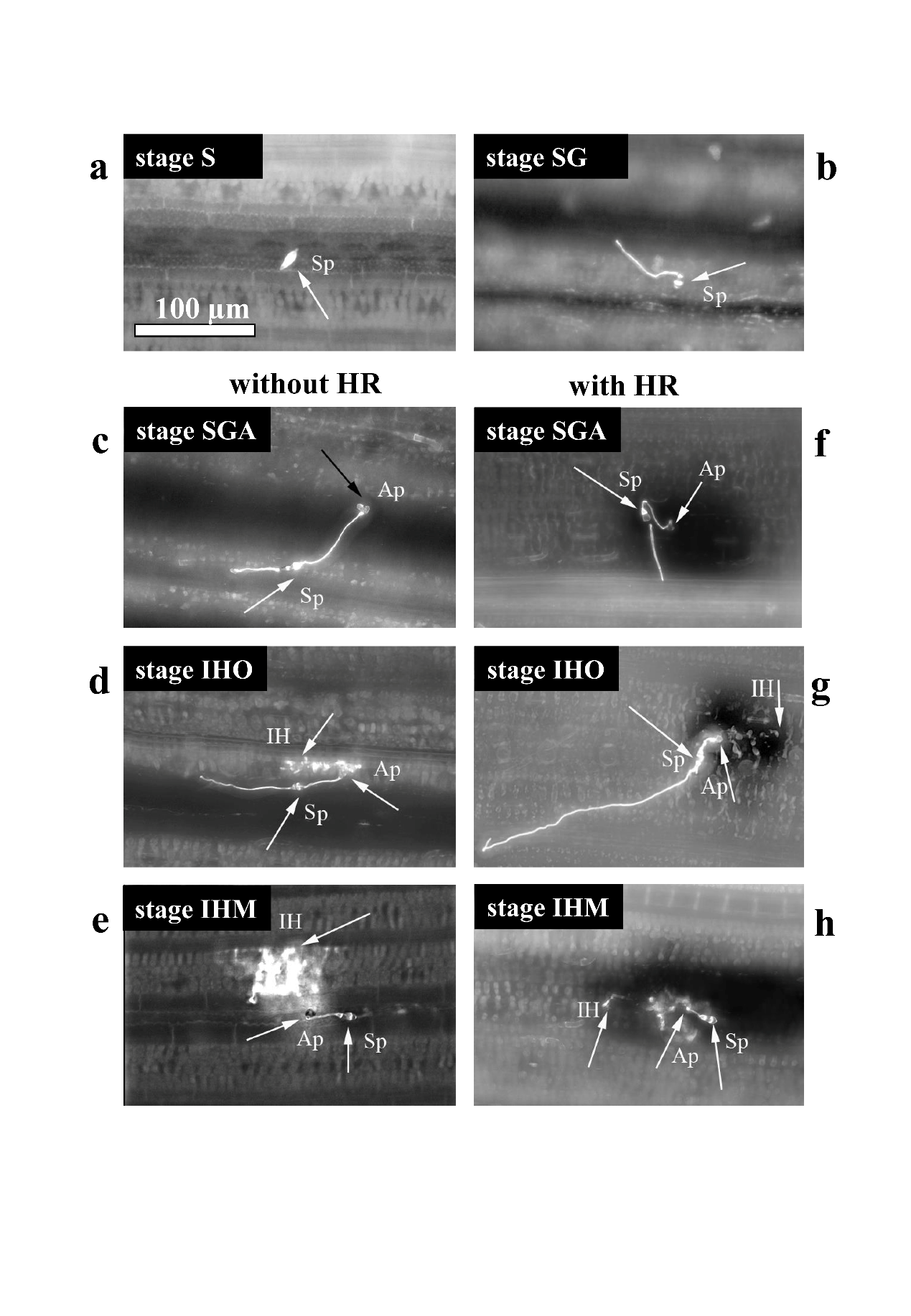


**Fig. S1** Representative images to illustrate the staging system used to classify the colonisation of plant tissue by including both strains GY11-EV and GY11-Avrpia. a-e. without host HR response; f-h. with host HR response. a. Spore without germination. b. Spore with germination; c. Spore with germ tube and appressorium formation but without host HR response; d. Invasive hyphae in one cell without host HR response; e. Invasive hyphae in multiple cells without host HR response; f. Spore with germ tube and appressorium formation with host HR response; g. Invasive hyphae in one cell with host HR response; h. Invasive hyphae in multiple cells with host HR response. Sp spore; Ap appressorium; IH invasive hypha; SGA germinated spore with appressorium; IHO invasive hyphae in one cell; IHM invasive hyphae in multiple cells.

**
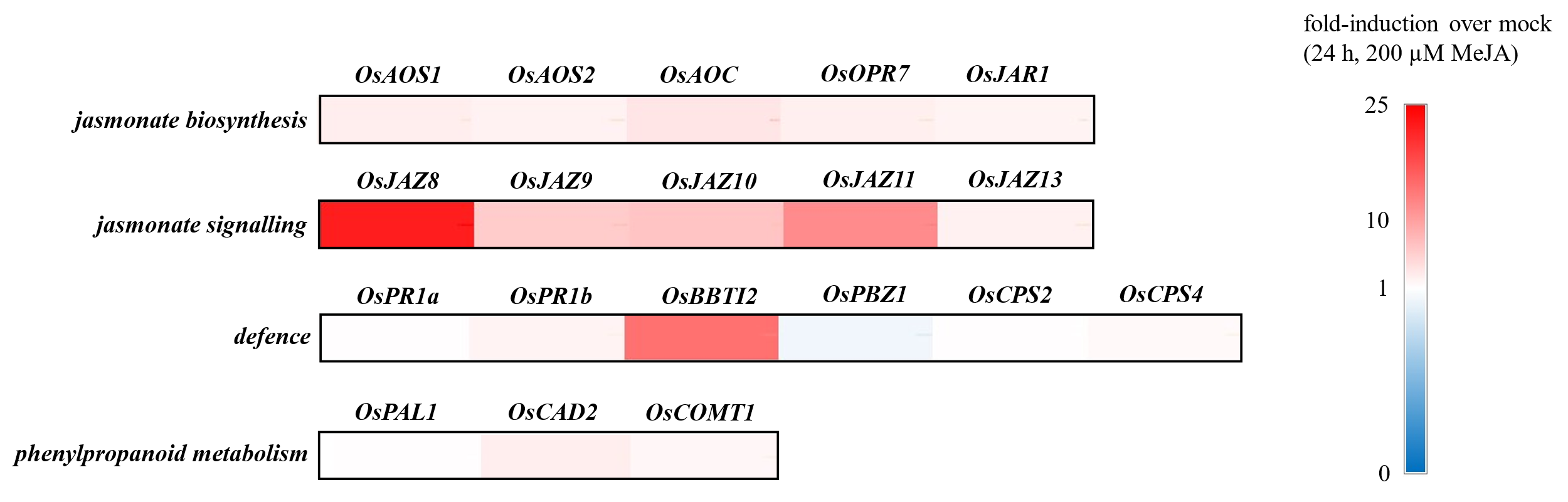
**

**Fig. S2** Steady-state transcript levels for genes of jasmonate biosynthesis, jasmonate signalling, defence, and phenylpropanoid metabolism in response to 200 µM of MeJA, scored 24 hours after the onset of the treatment in leaves of the wildtype. The heat map shows the fold-induction over the mock treatment.





**Fig. S3** Hierarchical clustering of the transcript levels for genes of jasmonate biosynthesis (**a**), signalling (**b**), both jasmonate biosynthesis and signalling (**c**), defence (**d**), phenylpropanoid metabolism (**e**), *OsAOS2*+*OsJAZ9*+defence+phenylpropanoid metabolism (**f**) and all tested genes (**g**) in response to mock treatment, or inoculation with the compatible strain GY11-EV, or the incompatible strain GY11-AvrPia in WT and the two jasmonate biosynthesis mutants. Note: numbers for the heatmap row names represent the following: **1**: 2 dpi-mock-WT, **2**: 2 dpi-mock-*cpm2*, **3**: 2 dpi-mock-*hebiba*, **4**: 2 dpi-GY11-WT, **5:** 2 dpi-GY11-*cpm2*, **6**: 2 dpi-GY11-*hebiba*, **7**: 2 dpi-GY11-AvrPia-WT, **8:** 2 dpi-GY11-AvrPia-*cpm2*, **9**: 2 dpi-GY11-AvrPia-*hebiba*, **10**: 3 dpi-mock-WT, **11**: 3 dpi-mock-*cpm2*, **12**: 3 dpi-mock-*hebiba*, **13**: 3 dpi-GY11-WT, **14:** 3 dpi-GY11-*cpm2*, **15**: 3 dpi-GY11-*hebiba*, **16**: 3 dpi-GY11-AvrPia-WT, **17:** 3 dpi-GY11-AvrPia-*cpm2*, **18**: 3 dpi-GY11-AvrPia-*hebiba*.

**References**

1. Cai Q, Yuan Z, Chen M, Yin C, Luo Z, Zhao X, Liang W, Hu J, Zhang D: Jasmonic acid regulates spikelet development in rice. *Nat Commun* 2014, 5:3476-3489.<https://doi.org/10.1038/ncomms4476>.

2. Jain M, Nijhawan A, Tyagi AK, Khurana JP: Validation of housekeeping genes as internal control for studying gene expression in rice by quantitative real-time PCR. *Biochem Biophys Res Commun* 2006, 345(2):646-651.<https://doi.org/10.1016/j.bbrc.2006.04.140>.

3. Mitsuhara I, Iwai T, Seo S, Yanagawa Y, Kawahigasi H, Hirose S, Ohkawa Y, Ohashi Y: Characteristic expression of twelve rice PR1 family genes in response to pathogen infection, wounding, and defense-related signal compounds. *Mol Genet Genomics* 2008, 279(4):415-427.<https://doi.org/10.1007/s00438-008-0322-9>.

4. Toyomasu T, Usui M, Sugawara C, Otomo K, Hirose Y, Miyao A, Hirochika H, Okada K, Shimizu T, Koga J *et al*: Reverse-genetic approach to verify physiological roles of rice phytoalexins: characterization of a knockdown mutant of OsCPS4 phytoalexin biosynthetic gene in rice. *Physiol Plant* 2014, 150(1):55-62.<https://doi.org/10.1111/ppl.12066>.

5. Grand X, Espinoza R, Michel C, Cros S, Chalvon V, Jacobs J, Morel J-B: Identification of positive and negative regulators of disease resistance to rice blast fungus using constitutive gene expression patterns. *Plant Biotechnol J* 2012, 10(7):840-850.<https://doi.org/10.1111/j.1467-7652.2012.00703.x>.

6. Shimizu T, Lin F, Hasegawa M, Okada K, Nojiri H, Yamane H: Purification and identification of naringenin 7-O-methyltransferase, a key enzyme in biosynthesis of flavonoid phytoalexin sakuranetin in rice. *J Biol Chem* 2012, 287(23):19315-19325.<https://doi.org/10.1074/jbc.M112.351270>.

7. Koshiba T, Hirose N, Mukai M, Yamamura M, Hattori T, Suzuki S, Sakamoto M, Umezawa T: Characterization of 5-hydroxyconiferaldehyde O-methyltransferase in *Oryza sativa*. *Plant Biotechnol* 2013, 30(2):157-167.<https://doi.org/10.5511/plantbiotechnology.13.0219a>.

8. Ookawa T, Inoue K, Matsuoka M, Ebitani T, Takarada T, Yamamoto T, Ueda T, Yokoyama T, Sugiyama C, Nakaba S *et al*: Increased lodging resistance in long-culm, low-lignin gh2 rice for improved feed and bioenergy production. *Sci Rep* 2014, 4:6567-6576.<https://doi.org/10.1038/srep06567>.

9. Shimizu T, Miyamoto K, Miyamoto K, Minami E, Nishizawa Y, Iino M, Nojiri H, Yamane H, Okada K: OsJAR1 contributes mainly to biosynthesis of the stress-induced jasmonoyl-isoleucine involved in defense responses in rice. *Biosci Biotechnol Biochem* 2013, 77(7):1556-1564.<https://doi.org/10.1271/bbb.130272>.

10. Ye H, Du H, Tang N, Li X, Xiong L: Identification and expression profiling analysis of TIFY family genes involved in stress and phytohormone responses in rice. *Plant Mol Biol* 2009, 71(3):291-305.<https://doi.org/10.1007/s11103-009-9524-8>.
